# Mapping and dynamic monitoring of desertification on the Qinghai-Tibetan Plateau using surface Albedo and Solar-Induced Chlorophyll Fluorescence

**DOI:** 10.1371/journal.pone.0359348

**Published:** 2026-09-25

**Authors:** Zhijian Zhao, Hui Lin, Lei Wu, Linling Tang, Xin Xiao

**Affiliations:** 1 College of Mathematics and Computer, Xinyu University, Xinyu, China; 2 Key Laboratory of Poyang Lake Wetland and Watershed Research Ministry of Education/Postdoctoral Research Station of Chemistry, Jiangxi Normal University, Nanchang, China; 3 The School of Geography and Environment, Jiangxi Normal University, Nanchang, China; Lanzhou Jiaotong University, CHINA

## Abstract

Desertification poses a significant threat to humanity’s long-term survival and sustainable development. In this study, four types of 2D desertification assessment models were created by integrating surface albedo with four vegetation-related indicators: NDVI, MSAVI, EVI, and solar-induced chlorophyll fluorescence (SIF). A comprehensive analysis of the spatiotemporal variations in desertification on the Qinghai-Tibetan Plateau (QTP) from 2001 to 2020 was then conducted. Among the four models, the SA-SIF model demonstrated the highest overall accuracy (0.8675; 95% CI: 0.8512–0.8838) and Kappa coefficient (0.8452; 95% CI: 0.8289–0.8615), significantly outperforming the other three models (*p* < 0.01, McNemar’s test). The SA-SIF model’s mapping results revealed substantial regional variability in desertification on the QTP, with the degree of desertification decreasing from northwest to southeast. Over the past decade, the expansion of severe and extremely severe desertification on the QTP has shown signs of slowing, although future trends may become more complex due to the interplay of climatic and anthropogenic factors. The SA-SIF model can serve as a reference for future desertification control operations on the QTP.

## 1. Introduction

Global climatic conditions have changed significantly over the last few decades. As global temperatures continue to rise, a range of ecological and environmental concerns have emerged [1–13], including floods [2], sea level rise [2,3], hydrological imbalance [4], coastal erosion [5], glacier melting [5], permafrost degradation [5,6], rapid carbon release [5–7], forest fires [8], urban heat island effects [9], and droughts [10–13]. Among these, drought is regarded as a high-risk and frequently occurring natural hazard [11–13]. Prolonged droughts can degrade plant ecosystems, trigger water resource crises, accelerate soil erosion, and ultimately induce desertification [11–15]. Desertification has devastating consequences for ecosystems, the natural environment, socioeconomic systems, and human health [14,15]. To safeguard the natural environment for future generations, land desertification must be accurately mapped and monitored.

The Qinghai-Tibetan Plateau (QTP) is often referred to as a “gene bank” and represents the world’s highest cold plateau. This vast region harbors a wide diversity of species [6,14,16], with complex hydrology and a fragile ecological system [11–17]. Ecological and climatic changes on the QTP affect global ecology and climate through atmospheric circulation, carbon cycling, and hydrological processes [11,12,16,18–20]. In recent decades, desertification on the QTP has intensified due to rising temperatures and decreasing precipitation [12,14,21]. This has caused glacier melting [12], permafrost deterioration [12,19], hydrological system disruption [11,12], extreme weather events [21], exposed land area growth [14,21], and widespread vegetation mortality [14,21]. Effective environmental management and restoration are therefore essential for human survival and progress. Understanding the temporal and spatial evolution of desertification on the QTP is crucial not only for the plateau itself but also for global desertification management efforts.

Currently, approaches for monitoring desertification on the QTP can be classified into two primary categories: indirect and direct monitoring. Indirect monitoring strategies generally emphasize drought circumstances, followed by a comprehensive examination of desertification based on ecological indices, soil characteristics, and human behavior. Common indicators for drought monitoring include the ecological water deficit index [22,23], crop water stress index [22], Palmer drought severity index [24,25], standardized soil moisture index [26,27], standardized precipitation index [27,28], groundwater drought index [29], standardized runoff index [30], standardized precipitation evapotranspiration index [31–33], and composite drought index [34]. Despite the specialized applications of these drought monitoring indices in various contexts, their estimations predominantly rely on precipitation, soil moisture content, and evapotranspiration. Indirect monitoring methods have been employed to examine the climatic parameters leading to desertification, based on the principle of hydrological imbalance [12]. However, in the absence of further data, this technique cannot precisely represent the fundamental attributes of desertification, including alterations in land and vegetation.

Direct and indirect monitoring methods differ from each other. Direct monitoring helps clarify the key traits of desertification. Desertification intensifies through flora deterioration and land exposure [34]; hence, direct monitoring methods focus on these changes. A single vegetation index was used to analyze vegetation changes. Most of them use the normalized differential vegetation index (NDVI) [35,36]. The NDVI characterizes vegetation using the near-infrared-red reflectance difference [35]. The NDVI calculation method is simple; however, background noise and air aerosol interference can cause significant vegetation coverage errors [37,38]. The modified soil-adjusted vegetation index (MSAVI) and enhanced vegetation index (EVI) were established to improve NDVI. The MSAVI has a dynamic soil adjustment. This reduces the effect of soil moisture and light on the computations [39]. EVI incorporates the blue spectral band and soil properties. This reduces soil-atmosphere interference [40]. MSAVI and EVI have helped NDVI overcome its limitations. However, these three vegetation markers may only show a basic structure. However, these models cannot accurately describe plant physiology. The solitary optical measure that directly represents the physiological state of vegetation is solar-induced chlorophyll fluorescence (SIF), unlike the other three vegetation indices. SIF is sensitive to plant photochemical efficiency and chlorophyll content. Plant physiological conditions can be determined instantly [41–43]. SIF can detect faint vegetative signals in arid and cold climates [42,43]. SIF helps to solve challenges caused by limited vegetation functional knowledge. All four measures were directly related to vegetation cover. Vegetation increases index values, whereas increasing desertification decreases them.

Tracking land changes is a crucial approach for evaluating the degree of desertification using direct monitoring techniques, in addition to monitoring flora alterations. Most conventional land change monitoring techniques do not incorporate vegetation indices and instead depend exclusively on the surface albedo (SA) for evaluation [44,45]. SA is a dimensionless variable. It denotes the ratio of solar shortwave radiation energy reflected from the Earth’s surface back into space to the total solar radiation energy incident upon the surface [46,47]. SA can provide details on soil properties, the effects of anthropogenic activities, and surface classifications [48,49]. The numerical range of SA spans from 0 to 1. Regions with lower SA values exhibit relatively dense vegetation, whereas areas with higher SA values show relatively sparse vegetation [50,51]. Consequently, SA values increase as desertification intensifies. It is generally recognized that the darkening of SA on the QTP drives vegetation greening [52,53], meaning that reduced SA promotes vegetation growth. This occurs because decreased SA increases net surface radiation and raises near-surface temperatures, alleviating low-temperature constraints and stimulating vegetation growth. Conversely, an increase in SA inhibits vegetation growth. Tian et al. demonstrated a negative correlation between SA and NDVI on the QTP [51]. In other words, there is a negative correlation between SA and four vegetation indices (NDVI, MSAVI, EVI, and SIF).

Traditional desertification monitoring methods typically rely on a single vegetation indicator or SA alone, making it difficult to comprehensively characterize both vegetation physiognomy and surface properties. To dynamically monitor desertification levels, vegetation and land-use changes must be examined in an integrated manner. In this project, four 2D desertification assessment models were constructed: SA-NDVI, SA-MSAVI, SA-EVI, and SA-SIF. The SA-SIF model emphasizes the relationship between the surface state and plant physiology, whereas the other three models evaluate the surface state in conjunction with plant structures. Using a threshold classification method, the differences in desertification detection among the four models on the QTP were assessed. These four model groups collectively form a desertification monitoring framework that better captures both “vegetation (structure/physiology) degradation” and “soil exposure.” This approach is expected to improve desertification monitoring and classification on the QTP, thereby supporting the development of the natural environment and the sustainable use of land resources in the region.

## 2. Methods

### 2.1. Study area

**Fig 1:**
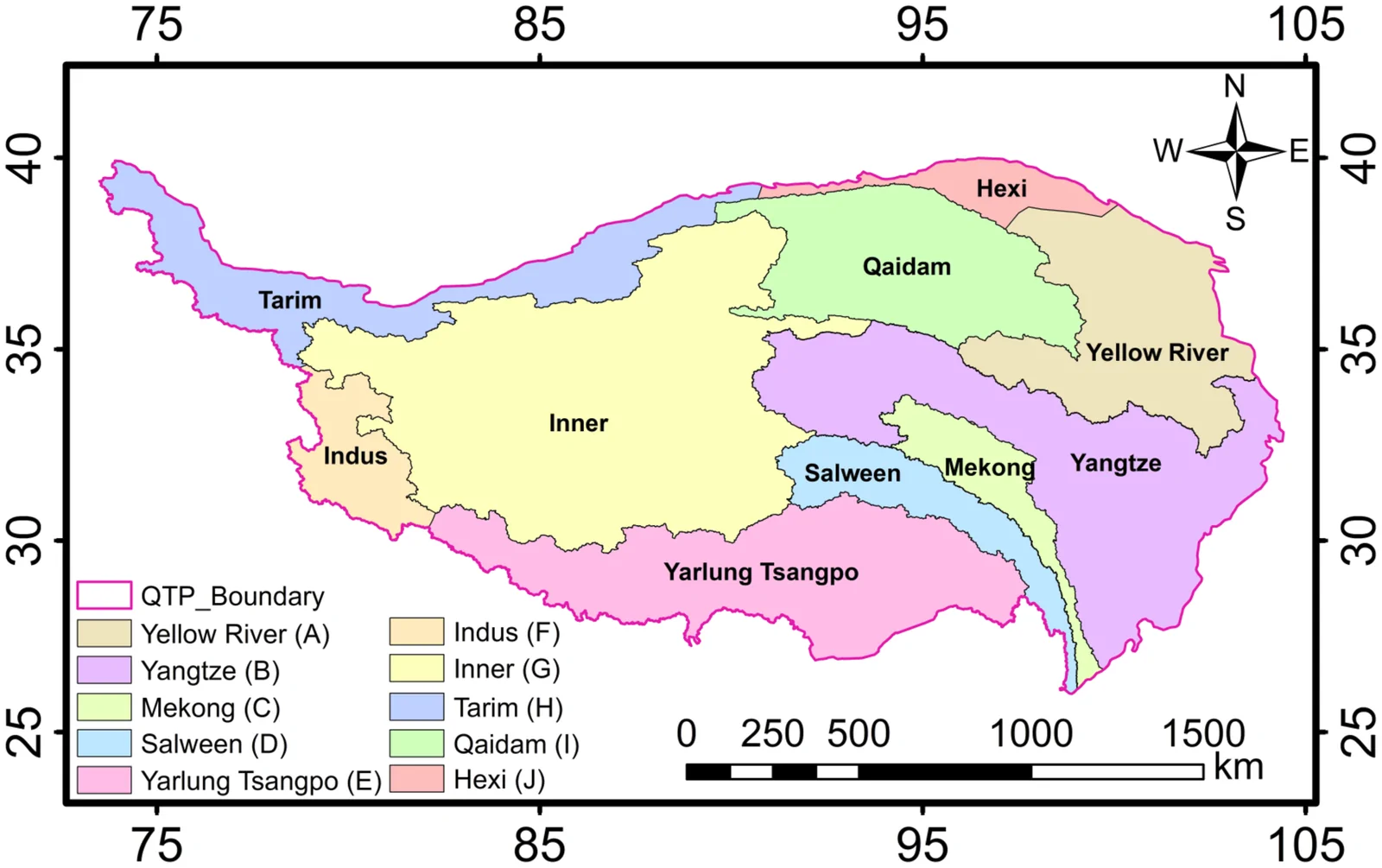
River basins on the QTP and their geographic expanse. The map encompasses ten principal basins: the Yellow River (A), Yangtze (B), Mekong (C), Salween (D), Yar-lung Tsangpo (E), Indus (F), Inner (G), Tarim (H), Qaidam (I), and Hexi (J). The map was generated via ArcGIS 10.8.2 (https://www.esri.com/en-us/arcgis/). The basemap and MODIS data were obtained from the NASA LP DAAC (https://lpdaac.usgs.gov/).

The QTP extends from the Pamir Plateau to the Hengduan Mountains, located in southwestern China at 26°00′–39°47′ N and 73°19′–104°47′ E [6,20]. It encompasses an extensive region that includes Qinghai, Tibet, and portions of Sichuan, Yunnan, Xinjiang, and Gansu. The QTP is characterized by extensive mountain ranges and a diverse array of lakes, as exemplified by Mount Everest and the Qinghai Lake. The terrain features lower elevations in the southeast and higher elevations in the northwest [6,20], resulting in unique geomorphological and climatic characteristics of the region. The QTP hosts multiple vegetation zones owing to climate heterogeneity, particularly vertical variations. The QTP showcases varied ecosystems with forests, scrub, meadows, grasslands, and deserts, from the snow line at high altitudes to grasses at low elevations. Fig 1 shows the study region and the distribution of major river basins on the QTP, delineated using ArcGIS 10.8.2. Fig 1 encompasses ten principal basins: Yellow River (A), Yangtze (B), Mekong (C), Salween (D), Yar-lung Tsangpo (E), Indus (F), Inner (G), Tarim (H), Qaidam (I), and Hexi (J).

### 2.2. Data sources and preprocessing

Six data types were employed in this study. First, MOD09A1 data (DOI: https://doi.org/10.5067/MODIS/MOD09A1.006 (accessed on 5 December 2024)) with 500 m spatial resolution in bands 1–7 were used for NDVI, EVI, and MSAVI extraction. Second, the National Tibetan Plateau Data Center (TPDC, https://data.tpdc.ac.cn) provides global continuous SIF data (DOI: https://doi.org/10.11888/Ecolo.tpdc.271751 (accessed on 5 December 2024)), covering 2000–2022 and generated via a neural network model. The data were subjected to sensor-to-sensor bias reduction and resampled to 500 m spatial resolution using bilinear interpolation. Third, MCD43A3 data (DOI: https://doi.org/10.5067/MODIS/MCD43A3.006 (accessed on 7 January 2025)) with sinusoidal projection provided daily 16-day BRDF model outputs at 500 m spatial resolution, including both black-sky and white-sky albedo. SA in this study was derived from MCD43A3 data. Fourth, Landsat 7 data were obtained from the Google Earth Engine (GEE) platform, comprising 8 bands: bands 1–5 and 7 (multispectral) at 30 m resolution, band 6 (thermal infrared) at 60 m resolution, and band 8 (panchromatic) at 15 m resolution, all with a 16-day temporal resolution. For validation, imagery from 2001 to 2020 on the QTP during the vegetation growth season (May–September) with cloud cover below 5% was selected. Fifth, meteorological data for the QTP were sourced from the TPDC. The dataset includes key climatic variables, including precipitation and air temperature, and wind speed (DOI: https://doi.org/10.11888/Atmos.tpdc.300470 (accessed on 5 December 2024)). Derived from daily observations spanning 1980–2020, the data were downscaled to 500 m resolution using the Anusplin interpolation method. This dataset was used to examine the climatic drivers of desertification dynamics in this study. Sixth, grazing intensity data for the QTP were also obtained from the TPDC (DOI: https://doi.org/10.11888/Ecolo.tpdc.271513 (accessed on 5 December 2024)). This dataset was generated by spatializing statistical livestock data from pastoral areas of the QTP through a multi-factor spatial model, yielding gridded grazing intensity layers (sheep units, MU/km²). The data cover 2000–2019 with a spatial resolution of 250 m. This dataset was employed to assess the role of anthropogenic grazing pressure in desertification processes. All preprocessing procedures were implemented on the GEE platform, including gap-filling, radiometric calibration, atmospheric correction, projection transformation, cropping, and mosaicking.

For Landsat 7 ETM+ imagery used in the validation, the SLC-off striping artifacts were addressed using the neighborhood similar pixel interpolator (NSPI) gap-filling algorithm [54]. This method fills missing data by identifying spectrally similar pixels within a local moving window and interpolating values based on their spectral similarity and spatial proximity. To ensure reliable validation, an initial pool of candidate points was selected using stratified random sampling across the study area, considering the SLC-off data gaps. S1 Fig shows the spatial distribution of the final 400 validation points across the QTP. After applying the NSPI gap-filling procedure, pixels with more than 50% of their values derived from gap-filling (i.e., > 50% missing data in the original scene) were excluded to ensure data quality. From the remaining gap-filled pixels, 400 high-quality pixels were retained as the final validation sample, forming the basis for the confusion matrices reported in S1–S4 Tables. The gap-filling process may introduce minor uncertainties in pixel-by-pixel matching; however, the exclusion of heavily gap-filled pixels and the use of independent validation samples minimize the potential impact on the accuracy assessment.

Although MCD43A3 data are widely applied for extracting SA, it remains unclear whether the widespread permafrost landscapes and freeze-thaw cycles across the QTP affect the applicability of these data. This study addresses this question through the following analyses. (a) At the algorithmic level, MCD43A3’s kernel-driven BRDF model dynamically adapts to surface roughness variations caused by freeze-thaw cycles in permafrost regions. Additionally, its built-in cloud and ice/snow removal algorithms provide preliminary handling for unique surface conditions in permafrost areas. (b) Previous research (Chen, C. et al., 2021) [53] demonstrated the applicability of MCD43A3 data on the QTP. (c) This study employs daily SA data with 100 m spatial resolution from 2002 to 2022 as reference, conducting stratified validation according to freeze-thaw phases (freezing period, thawing period, and stable period). The reference data were obtained from the TPDC (DOI: https://doi.org/10.11888/Terre.tpdc.272047 (accessed on 5 December 2024)), which incorporates topographic effects of the QTP and exhibits high accuracy and spatio-temporal continuity. Table 1 shows that the minimum R² value for SA extraction across all phases is 0.8843, with a maximum RMSE of 0.0232 and maximum MAE of 0.0165. These small errors meet the experimental requirements.

**Table 1:** Stratified validation results for MCD43A3 SA across different freeze-thaw phases.

Freeze-thaw phase	Sample size	R²	RMSE	MAE
**Freezing period (November–March)**	151	0.9025	0.0214	0.0152
**Thawing period (April–May)**	61	0.8843	0.0232	0.0165
**Stable period (June–October)**	153	0.9178	0.0183	0.0137
**Overall (all phases)**	365	0.9036	0.0207	0.0154

### 2.3. Extraction of four vegetation indices

The NDVI equation is presented below [35,40]:


$$\begin{array}{c} NDVI=\frac{\left({Nir}_{B}-{Red}_{B}\right)}{\left({Nir}_{B}+{Red}_{B}\right)} \end{array}$$ (1)

The MSAVI equation is presented below [39]:


$$\begin{array}{c} MSAVI=\frac{\text{1}}{\text{2}}\left(\left(\text{2}{Nir}_{B}+\text{1}\right)-\sqrt{{\left(\text{2}{Nir}_{B}+\text{1}\right)}^{\text{2}}-\text{8}\left({Nir}_{B}-{Red}_{B}\right)}\right) \end{array}$$ (2)

where *Nir*_*B*_ is the surface reflectance in the near-infrared band (Band 2 of MOD09A1), and *Red*_*B*_ is the surface reflectance in the red band (Band 1 of MOD09A1).

The EVI equation is presented below [40]:


$$\begin{array}{c} EVI=G_{B}\frac{{Nir}_{B}-{Red}_{B}}{{Nir}_{B}+\;{C\text{1}}_{B}\times {Red}_{B}-{C\text{2}}_{B}\times {Blue}_{B}+L_{B}} \end{array}$$ (3)

where *G*_*B*_ = 2.5 represents the gain factor. *C1*_*B*_ = 6 and *C2*_*B*_ = 7.5 are coefficients used to adjust for atmospheric aerosol scattering. *L*_*B*_ possesses a starting value of 1 to compensate for canopy and soil background. *Blue*_*B*_ represents the surface reflectance in the blue band (Band 3 of MOD09A1).

SIF is a dimensioned fluorescence intensity, whereas NDVI, MSAVI, and EVI are all dimensionless indices. SIF exhibits significant differences in both dimensions and numerical ranges compared to these three types of vegetation indices. To eliminate dimensional differences and achieve multi-variable comparability, Min-Max normalization (deviation standardization) [55] was employed in this study. The core logic of this method is to map variables with different ranges to a unified interval, thereby preventing analysis weight imbalances caused by dimensional differences. Simultaneously, it precisely matches the typical ranges of vegetation indices, better aligning with the coupling analysis requirements between SIF and vegetation indices. The normalized SIF is calculated as follows:


$$\begin{array}{c} {SIF}_{Nor}=\left(\frac{SIF-{SIF}_{Min}}{{SIF}_{Max}-{SIF}_{Min}}\right)\times \left({VI}_{Max}-{VI}_{Min}\right)+{VI}_{Min} \end{array}$$ (4)

The normalization parameters were determined based on statistical analysis of study area data. Here, *SIF*_*Min*_ = 0 corresponds to areas with no vegetation, while *SIF*_*Max*_ = 30 represents the peak value for areas with high vegetation coverage. Both *SIF*_*Min*_ and *SIF*_*Max*_ were measured in mWm^–2^ sr^–1^ nm^–1^. The values of *VI*_*Min*_ and *VI*_*Max*_ were –0.2 and 0.9, respectively, corresponding to the target vegetation index range. These values originate from the average extreme ranges of the three vegetation indices: NDVI, MSAVI, and EVI.

SIF anomalies may occur owing to factors such as snow cover, permafrost thawing, and cloud interference on the QTP, necessitating the identification and handling of SIF outliers. The 3σ principle [56] was adopted in this study to identify SIF anomalies. This principle is based on the theoretical assumption that SIF data follow an approximate normal distribution (Shapiro-Wilk test [57]), with 99.7% of values falling within the mean ±3σ range, while values exceeding this range are considered anomalies. Subsequently, the identified anomalies were processed using the neighborhood pixel time series interpolation method [58]. This method is grounded in the spatiotemporal continuity of vegetation growth in the QTP, where adjacent pixels or temporal SIF values exhibit strong correlations. Interpolation eliminates anomalies while preserving the natural dynamic trends of SIF.

### 2.4. Calculation of SA

Liang’s proposed approach [59,60] for SA has high accuracy. This approach was employed to compute the SA using the subsequent equation [59,60]:


$$\begin{array}{c} SA=\text{0}.\text{1}\text{6}\text{0}\beta_{\text{1}}+\text{0}.\text{2}\text{9}\text{1}\beta_{\text{2}}+\text{0}.\text{2}\text{4}\text{3}\beta_{\text{3}}+\text{0}.\text{1}\text{1}\text{6}\beta_{\text{4}}+\text{0}.\text{1}\text{1}\text{2}\beta_{\text{5}}+\text{0}.\text{0}\text{8}\text{1}\beta_{\text{7}}-\text{0}.\text{0}\text{0}\text{1}\text{5} \end{array}$$ (5)

where –0.0015 serves as a coefficient for offset correction, applicable for the elimination of residual errors, including atmospheric corrections. *β*_*1*_, *β*_*2*_, *β*_*3*_, *β*_*4*_, *β*_*5*_, and *β*_*7*_ correspond to bands 1–5 and band 7 of the MCD43A3 data product, respectively.

The obtained SA is then normalized using the following equation:


$$\begin{array}{c} {SA}_{Nor}=\left(\frac{SA-{SA}_{Min}}{{SA}_{Max}-{SA}_{Min}}\right) \end{array}$$ (6)

where *SA*_*Min*_ and *SA*_*Max*_ are the minimum and maximum values of SA on the QTP.

### 2.5. Development of four 2D desertification assessment models (SA-NDVI, SA-MSAVI, SA-EVI, and SA-SIF)

**Fig 2:**
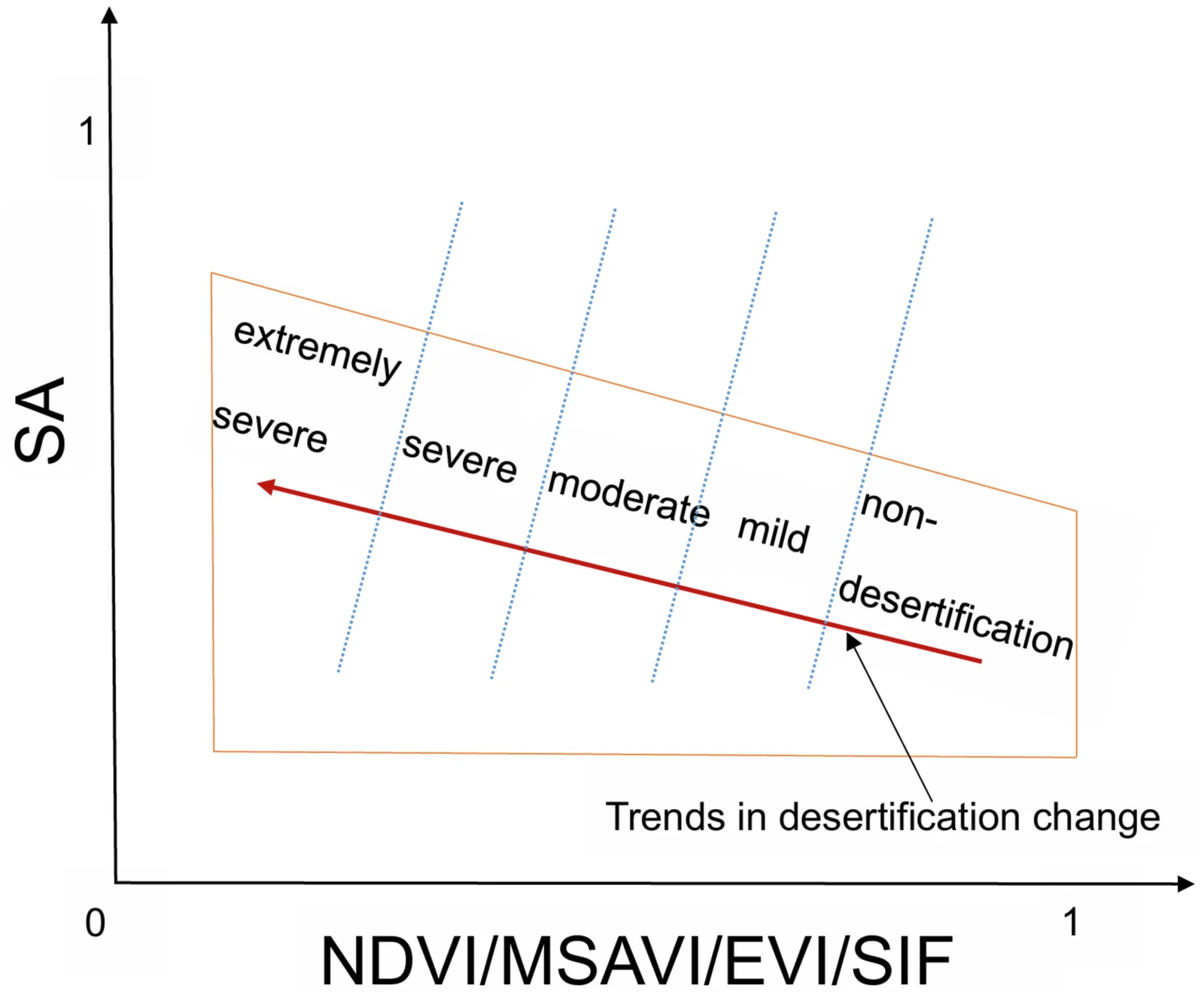
Four groups of 2D desertification assessment models, SA-NDVI, SA-MSAVI, SA-EVI, and SA-SIF. Based on the trend of land desertification, the categories are arranged from right to left as follows: non-desertification, mild desertification, moderate desertification, severe desertification, and extremely severe desertification.

The specific steps for constructing four groups of 2D desertification assessment models in this study were as follows:

(a) The four vegetation indices (NDVI, MSAVI, EVI, and SIF) and SA data were reconstructed on the TIMESAT platform using the SG approach [61], eliminating noise that deviated from the standard trajectory.
(b) Annual data for the four vegetation indices were obtained using the maximum value composite technique.
(c) Annual data extraction for SA using the minimum value compositing technique.
(d) The 2,000 sample points were randomly generated within the study area using ArcGIS 10.8.2.
(e) Extraction of four vegetation indices and SA at 2000 sample points.
(f) Four sets of scatter plots were constructed for the regression analysis, with SA as the vertical axis and the four vegetation indices as the horizontal axis. The trend of these scatter plots represents the desertification trajectories.
(g) Based on the framework of Verstraete and Pinty [62], four groups of 2D desertification assessment models, namely SA-NDVI, SA-MSAVI, SA-EVI, and SA-SIF, were delineated as illustrated in Fig 2 Dividing the feature space vertically according to the desertification gradient allows for the effective differentiation of desertified terrain at varying degrees.

(h) Based on the gradient of change and the developmental process of land desertification, the desertification classes depicted in Fig 2 were categorized into five levels: non-desertification, mild desertification, moderate desertification, severe desertification, and extremely severe desertification. To facilitate an objective comparison across the four model groups, a standardized grading system was established using the natural breaks (Jenks) method [63,64] in ArcGIS 10.8.2. This method maximizes the between-category differences while minimizing the within-category disparities.
(i) The vertical position in each feature space can be fitted using the following linear binary equation:

$$\begin{array}{c} D_{I}=\theta \times VIF-{SA}_{Nor} \end{array}$$ (7)

where *D*_*I*_ represents the desertification differentiation index, *VIF* represents the four vegetation indices (NDVI, MSAVI, EVI, and SIF), and *θ* is a constant determined from the slopes of the four feature spaces shown in Fig 2.

Parameter *θ* in equation (7) represents the slope of the linear relationship between the scaled vegetation index (*VIF*) and SA in the 2D feature space. Physically, *θ* serves as a scaling factor that normalizes the vegetation signal, which captures photosynthetic activity or greenness**,** to the same dynamic range as the albedo signal, which captures the surface reflectance properties. This normalization is essential because *VIF* and SA have different units and magnitudes; without scaling, their linear combination would be dominated by the variable with a larger numerical range.

Ecologically, the subtraction of *SA*_*Nor*_ from *θ × VIF* is grounded in the bidirectional nature of desertification: as land degrades, the vegetation cover and photosynthetic activity decline (decreasing *VIF*), while bare soil exposure increases albedo (increasing SA). The difference between the scaled vegetation signal and the normalized albedo therefore represents the net “desertification signal.” Larger positive values indicate healthier vegetation cover, while negative values indicate degradation.

To assess the sensitivity of *D*_*I*_ to variations in *θ*, a sensitivity analysis was conducted by perturbing *θ* by ±10% and ±20% from its estimated value. The resulting *D*_*I*_ values showed minimal variation (coefficient of variation < 5%), indicating that the model is robust to reasonable uncertainties in the *θ* estimation.

(j) Hierarchical mapping of desertification in the study area was performed using the desertification differentiation indices from each of the four models.

### 2.6. Accuracy assessment of desertification classification

The specific steps for evaluating the accuracy of the desertification classification models in this study are as follows:

(a) The validation data were derived from Landsat 7 imagery covering the entire 2001–2020 study period, with reference interpretations for each year based on the best available cloud-free imagery during the growing season. This ensures that the validation is not limited to a single reference year but rather encompasses the full temporal range of the study. To ensure the representativeness and statistical robustness of the validation, a stratified random sampling strategy was adopted. Specifically, the QTP was stratified by two criteria: (1) desertification grade, consisting of five classes (non-desertification, mild, moderate, severe, and extremely severe), and (2) geographic sub-basin, comprising the ten major hydrological basins shown in Fig 1 The 400 validation points were generated using the Create Accuracy Assessment Points tool in ArcGIS 10.8.2 with stratified random sampling, where the number of points allocated to each stratum was proportional to the area of that stratum within the QTP. Importantly, this is an area-weighted allocation rather than an equal-number allocation across strata. This study did not adopt a balanced stratification scheme with equal sample sizes per stratum, as such a scheme would over-represent small-area strata and under-represent larger desertification regions. To justify the sample size of 400 points, a power analysis was conducted for the classification accuracy assessment. Assuming a desired precision of ±5% at the 95% confidence level and an expected overall accuracy of 85%, the minimum required sample size was calculated as 196 points. The sample size of 400 points therefore provides more than twice the minimum requirement, ensuring sufficient statistical power to reliably validate the classification accuracy across the vast and heterogeneous QTP. S1 Fig shows the spatial distribution of the 400 validation points.
(b) Using Landsat 7 high-resolution image interpretation results as the ground-truth reference, pixel-by-pixel matching was performed between the desertification classification results of the four models and Landsat 7 interpretations. Multiclass confusion matrices were constructed for each model (S1–S4 Tables).
(c) The validation points were strictly independent of the samples used to construct the feature space models. For model calibration, a separate set of 400 training samples was generated using the Create Random Points tool in ArcGIS 10.8.2 through simple random sampling across the entire QTP, without stratification or area-weighted allocation. These two sample sets were generated independently: the training samples were used exclusively for constructing the 2D feature spaces and calibrating model parameters (*θ* values and *D*_*I*_ classification thresholds), whereas the validation points were reserved solely for final accuracy assessment. The training and validation samples did not share any spatial locations and were collected from the same time period (2001–2020) but with strictly non-overlapping pixel positions, as confirmed by spatial overlay checks in ArcGIS 10.8.2. No validation point was used, directly or indirectly, in any stage of model training, parameter calibration, or threshold determination. The validation set was only applied after all model parameters had been finalized, thereby completely preventing data leakage and ensuring an unbiased evaluation of classification accuracy.
(d) Based on the constructed confusion matrices, the classification accuracy was evaluated using four metrics: user accuracy, producer accuracy, overall accuracy, and Kappa coefficient.

To determine whether the observed performance differences among the four models were statistically significant, 95% confidence intervals for overall accuracy and Kappa coefficients were calculated using bootstrap resampling with 1,000 iterations. Pairwise comparisons of classification accuracy were conducted using McNemar’s test [65].

The four metrics are as follows:

(a) User accuracy reflects the reliability of classification results [66], indicating the proportion of pixels predicted to belong to a desertification category that actually belongs to that category.
(b) Producer accuracy focuses on the completeness of the classification model in identifying true categories [66], indicating the proportion of pixels that belong to a desertification category and are correctly classified.
(c) Overall accuracy measures the precision of the classification as a whole, and is defined as the proportion of pixels where the predicted result matches the true result of the total number of pixels evaluated [66].
(d) The Kappa coefficient is a consistency metric that excludes random classification interference [66], used to quantify the degree of agreement between the model predictions and actual results. Its value ranges from –1–1, with values closer to 1 indicating stronger agreement between the two sets of results. The calculation formula is as follows [66]:

$$\begin{array}{c} KC=\frac{\left(OA-K_{e}\right)}{\left(\text{1}-K_{e}\right)} \end{array}$$ (8)

$$\begin{array}{c} K_{e}=\frac{\sum_{i=\text{1}}^{n}\left(T_{i}\times K_{i}\right)}{N^{\text{2}}} \end{array}$$ (9)

where *OA* denotes overall accuracy, *K*_*e*_ represents the expected accuracy of random classification, *T*_*i*_ is the total number of true pixels in class *i*, *K*_*i*_ is the total number of predicted pixels in class *i*, *N* and *n* denote the total number of evaluated pixels (400) and the fixed number of classes (5), respectively.

### 2.7. Statistical analysis

To quantify the uncertainty of the accuracy estimates, the bootstrap method with 1,000 resampling iterations [67] was employed. For each bootstrap sample (drawn with replacement from the original 400 validation pixels), the overall accuracy and Kappa coefficient were computed. The bootstrap estimate of overall accuracy was calculated as follows [67]:


$$\begin{array}{c} {OA}_{boot}=\frac{\text{1}}{M}\sum_{m=\text{1}}^{M}{OA}_{m} \end{array}$$ (10)

where *M* = 1,000 is the number of bootstrap iterations, and *OA*_*m*_ denotes the overall accuracy computed from the *m*-th bootstrap sample. The standard error and 95% confidence intervals were derived from the bootstrap distribution of the accuracy estimates.

To determine whether the observed differences in classification performance among the four models were statistically significant, McNemar’s test [65] was applied for pairwise comparisons. The test statistic with continuity correction was calculated as follows [65]:


$$\begin{array}{c} \chi^{\text{2}}=\frac{{\left(|a_{wq}-a_{qw}|-\text{1}\right)}^{\text{2}}}{a_{wq}+a_{qw}} \end{array}$$ (11)

where *a*_*wq*_ denotes the number of pixels correctly classified by model *w* but misclassified by model *q*, and *a*_*qw*_ denotes the number of pixels correctly classified by model *q* but misclassified by model *w*. The statistic follows a chi-square distribution with one degree of freedom [68]. A test statistic exceeding 3.84 indicates a statistically significant difference at the 95% confidence level [68].

To quantitatively evaluate the relationships between desertification dynamics and potential driving factors, Pearson’s correlation analysis [69] was performed between the annual average *D*_*I*_ and key climatic variables (annual precipitation, annual mean temperature, and growing season temperature), as well as livestock density. The Pearson’s correlation coefficient was calculated as follows [69]:


$$\begin{array}{c} R=\frac{\sum_{t=\text{1}}^{T}\left({CM}_{t}-{CM}_{A}\right)\left(D_{It}-D_{IA}\right)}{\sqrt{\sum_{t=\text{1}}^{T}{\left({CM}_{t}-{CM}_{A}\right)}^{\text{2}}\cdot \sum_{t=\text{1}}^{T}{\left(D_{It}-D_{IA}\right)}^{\text{2}}}} \end{array}$$ (12)

where *CM*_*t*_ represents the climatic or anthropogenic variable for year *t*, *D*_*It*_ represents the annual average *D*_*I*_ for year *t*, *T* = 20 (2001–2020), *CM*_*A*_ denotes the mean of the climatic or anthropogenic variable over the study period, and *D*_*IA*_ denotes the overall mean of *D*_*I*_ over the study period. The significance of each correlation was tested using a two-tailed t-test [70].

## 3. Results

### 3.1. Spatial distribution of features of SA-NDVI, SA-MSAVI, SA-EVI and SA-SIF

**Fig 3:**
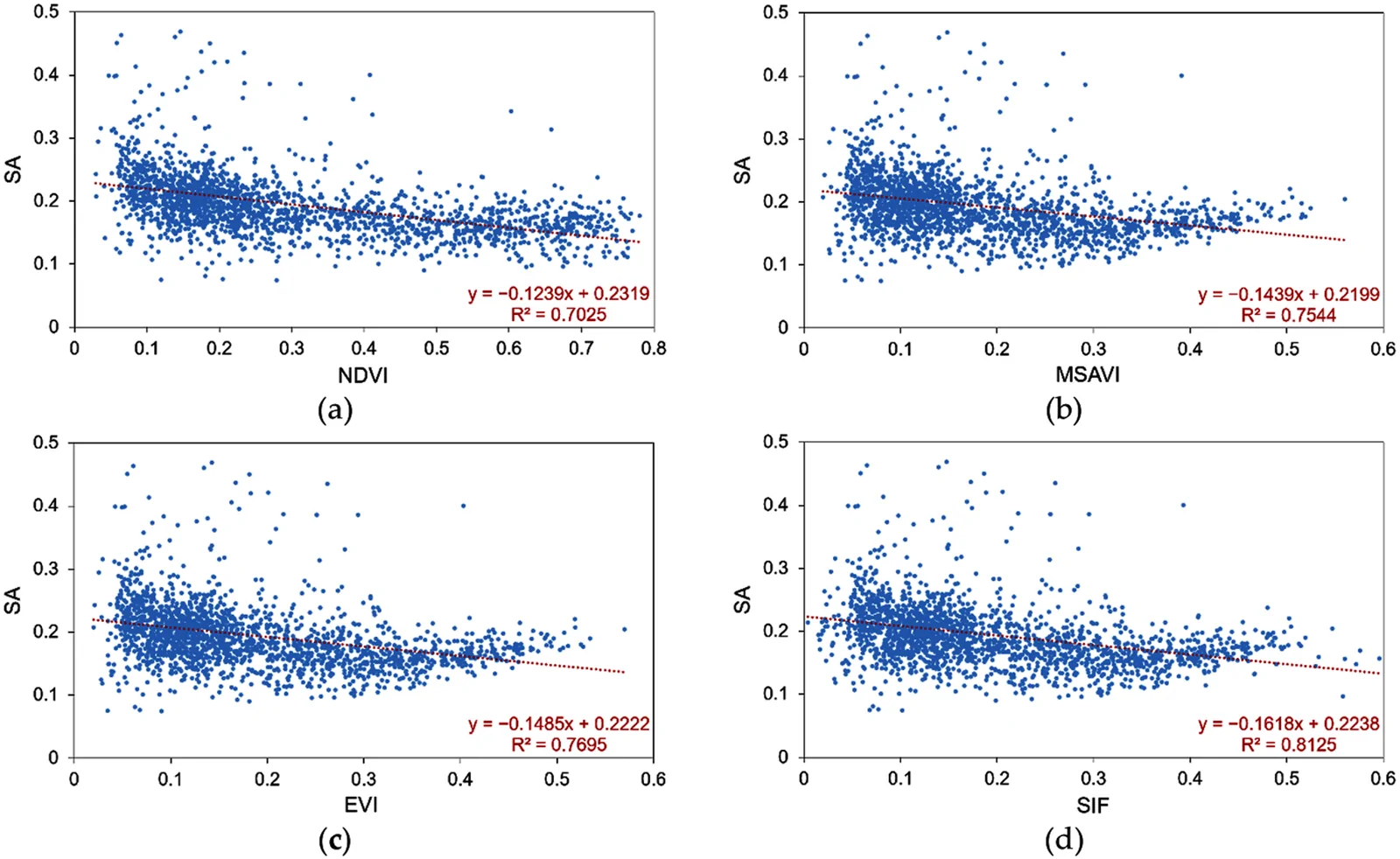
Spatial distribution of 2D features of (a) SA-NDVI, (b) SA-MSAVI, (c) SA-EVI, and (d) SA-SIF on the QTP in 2014.

Fig 3 shows the feature space scatter plots of SA-NDVI, SA-MSAVI, SA-EVI, and SA-SIF. The horizontal axis range of the NDVI group (0–0.8) was substantially wider than those of the other three groups, while the other three groups exhibited better continuity in spatial features. The SA-SIF group performed best in terms of minimum and maximum value identification. The slopes of the feature spaces for SA-NDVI, SA-MSAVI, SA-EVI, and SA-SIF were –0.1239, –0.1439, –0.1485, and –0.1618, respectively. SIF showed the highest correlation with SA, while NDVI showed the lowest. Accordingly, the *θ* values for these four groups in equation (7) were 8.0710, 6.9493, 6.7340, and 6.1805, respectively.

### 3.2. *D*_*I*_ classification of SA-NDVI, SA-MSAVI, SA-EVI, and SA-SIF spatial features

The four groups of *θ* obtained in subsection 3.1 were substituted into equation (7). The *D*_*I*_ grading for the four groups (SA-NDVI, SA-MSAVI, SA-EVI, and SA-SIF), derived using the natural breaks (Jenks) method [63,64], is presented in Table 2.

**Table 2:** *D*_*I*_ classification table for SA-NDVI, SA-MSAVI, SA-EVI, and SA-SIF spatial features.

Categories	SA-NDVI	SA-MSAVI	SA-EVI	SA-SIF
**Extremely severe**	*D*_*I*_ ≤ 0.0	*D*_*I*_ ≤ –0.1	*D*_*I*_ ≤ –0.05	*D*_*I*_ ≤ 0.1
**Severe**	0.0 < *D*_*I*_ ≤ 1.5	–0.1 < *D*_*I*_ ≤ 0.8	–0.05 < *D*_*I*_ ≤ 0.9	0.1 < *D*_*I*_ ≤ 1.0
**Moderate**	1.5 < *D*_*I*_ ≤ 3.0	0.8 < *D*_*I*_ ≤ 2.0	0.9 < *D*_*I*_ ≤ 2.2	1.0 < *D*_*I*_ ≤ 1.8
**Mild**	3.0 < *D*_*I*_ ≤ 4.5	2.0 < *D*_*I*_ ≤ 3.0	2.2 < *D*_*I*_ ≤ 3.0	1.8 < *D*_*I*_ ≤ 2.5
**Non-Desertification**	*D*_*I*_ > 4.5	*D*_*I*_ > 3.0	*D*_*I*_ > 3.0	*D*_*I*_ > 2.5

### 3.3. Hierarchical mapping of desertification on the QTP utilizing four 2D models

**Fig 4:**
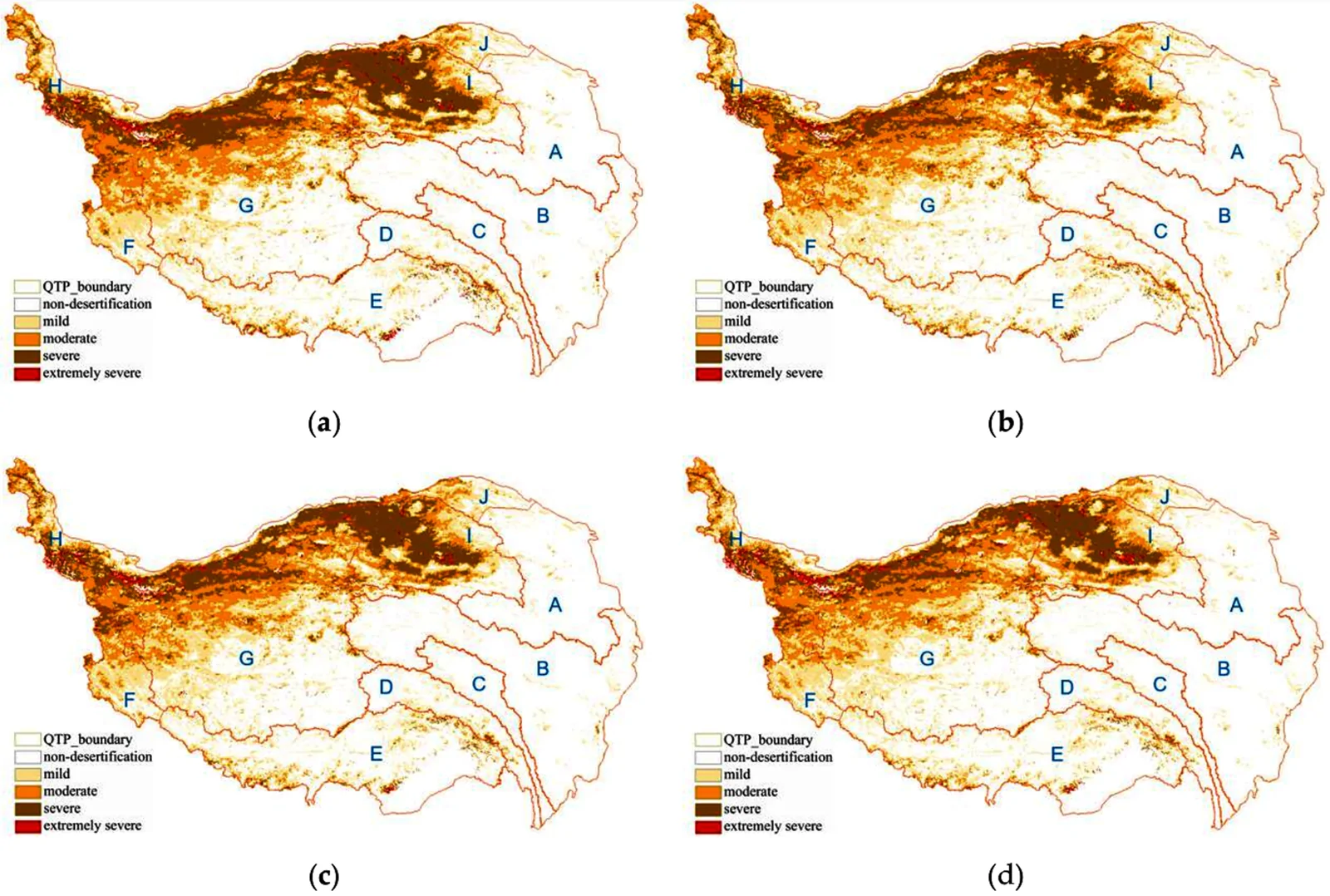
Classification mapping findings of desertification for four groups of 2D models on the QTP in 2014: (a) SA-NDVI, (b) SA-MSAVI, (c) SA-EVI, and (d) SA-SIF. The five levels of desertification are non-desertification, mild, moderate, severe, and extremely severe, sequentially. Maps were generated via ArcGIS 10.8.2 (https://www.esri.com/en-us/arcgis/). The basemap and MODIS data were obtained from the NASA LP DAAC (https://lpdaac.usgs.gov/). (Note: Other unlabeled geographic data remains consistent with Fig 1.).

The results in Fig 4 show that: (a) Extremely severe desertification is mainly found in zones H and I, with a small amount in zones E and F. These zones are mostly unvegetated saline and stony areas. (b) Severe desertification is mainly found in zones H and I, and to a lesser extent in zones F and G. These zones are mostly unvegetated saline, stony, gravelly, and sandy areas. (c) Moderate desertification is mainly found in the northern part of zones F and G and along the edges of zones H and I. These zones include vegetated earthy, sandy, and stony lands in addition to the unvegetated land types mentioned above. (d) Mild desertification is mainly distributed in zones D and E, the central part of zone G, the southern part of zone F, the edges of zones H and I, and the eastern part of zone J. The land types in these areas are predominantly mountainous. (e) Non-desertification is mainly found in the central and eastern parts of the QTP.

### 3.4. Validation and evaluation of desertification grading outcomes for four groups of 2D models

Table 3 presents the graded category accuracy (user accuracy/producer accuracy), overall accuracy, and Kappa coefficients for the four groups of 2D models. According to the data shown in Table 3, except for the producer accuracy for extremely severe desertification, the hierarchy of accuracy for each item is as follows: SA-SIF > SA-MSAVI > SA-EVI > SA-NDVI. Among these, the SA-SIF model exhibited the highest consistency with the interpretation results from Landsat 7 high-resolution imagery.

**Table 3:** Hierarchical category accuracy, overall accuracy, and Kappa coefficient for four groups of 2D models.

Group Name	Non- Desertification (U/P)	Mild (U/P)	Moderate (U/P)	Severe (U/P)	Extremely Severe (U/P)	OA	KC
**SA-NDVI**	0.8718/ 0.8831	0.7558/ 0.7738	0.7473/ 0.7473	0.7358/ 0.7573	0.6383/ 0.6667	0.7725	0.7346
**SA-MSAVI**	0.9231/ 0.9351	0.8095/ 0.8095	0.7912/ 0.7912	0.7885/ 0.7961	0.7778/ 0.7778	0.8225	0.7938
**SA-EVI**	0.8974/ 0.9091	0.7765/ 0.7857	0.7582/ 0.7582	0.7524/ 0.7670	0.6458/ 0.6889	0.7875	0.7521
**SA-SIF**	0.9740/ 0.9740	0.8889/ 0.8571	0.8571/ 0.8571	0.9000/ 0.8738	0.8000/ 0.7111	0.8675	0.8452

Note: U/P denotes user accuracy/producer accuracy, OA denotes overall accuracy, and KC denotes Kappa coefficient. The U/P accuracy is equal for the “Moderate Desertification” category in the table, because this category has distinct land cover characteristics, primarily defined by “moderate vegetation cover + intermediate SA,” corresponding to grasslands or sparsely vegetated areas; it neither represents high vegetation cover areas along riverbanks nor bare soil regions inland, exhibiting strong surface cover homogeneity with minimal mixed-pixel interference.

To quantify the uncertainty of these accuracy estimates, 95% confidence intervals for overall accuracy and Kappa coefficients were calculated using bootstrap resampling (1,000 iterations) [67]. The SA-SIF model achieved an overall accuracy of 0.8675 (95% CI: 0.8512–0.8838) and a Kappa coefficient of 0.8452 (95% CI: 0.8289–0.8615). Pairwise comparisons using McNemar’s test [65] confirmed that the overall accuracy of the SA-SIF model was significantly higher than that of SA-NDVI (0.7725, 95% CI: 0.7523–0.7927; *p* < 0.01), SA-MSAVI (0.8225, 95% CI: 0.8041–0.8409; *p* < 0.05), and SA-EVI (0.7875, 95% CI: 0.7682–0.8068; *p* < 0.01). The detailed results of bootstrap confidence intervals, McNemar’s tests, and Pearson correlation analyses are summarized in Tables 4–6, respectively. Table 4 shows that the SA-SIF model yielded the narrowest confidence intervals for both overall accuracy and Kappa coefficient. Table 5 confirms that all pairwise McNemar’s tests between SA-SIF and the other models were statistically significant. Table 6 reveals that growing season temperature exhibited the strongest correlation with *D*_*I*_ among the tested climatic variables.

**Table 4:** Bootstrap confidence intervals for overall accuracy and Kappa coefficients of the four models.

Model	Metric	Bootstrap Estimate	95% Confidence Interval
**SA-NDVI**	OA	0.7725	[0.7523, 0.7927]
**SA-NDVI**	KC	0.7346	[0.7112, 0.7580]
**SA-MSAVI**	OA	0.8225	[0.8041, 0.8409]
**SA-MSAVI**	KC	0.7938	[0.7724, 0.8152]
**SA-EVI**	OA	0.7875	[0.7682, 0.8068]
**SA-EVI**	KC	0.7521	[0.7298, 0.7744]
**SA-SIF**	OA	0.8675	[0.8512, 0.8838]
**SA-SIF**	KC	0.8452	[0.8289, 0.8615]

Note: Bootstrap resampling with 1,000 iterations was used to calculate 95% confidence intervals.

**Table 5:** McNemar’s test results for pairwise comparisons between SA-SIF and the other three models.

Comparison	*χ*^*2*^ Statistic	*p*-value
**SA-SIF vs. SA-NDVI**	18.24	< 0.01
**SA-SIF vs. SA-MSAVI**	5.76	< 0.05
**SA-SIF vs. SA-EVI**	12.96	< 0.01

Note: McNemar’s test with continuity correction. The test statistic follows a *χ²* distribution with 1 degree of freedom. A test statistic > 3.84 indicates statistical significance at the 95% confidence level.

**Table 6:** Pearson correlation analysis between annual average *D*_*I*_ and climatic and anthropogenic variables (2001–2020).

Variable	Pearson Correlation Coefficient (*r*)	*p*-value
**Annual Precipitation**	0.52	< 0.05
**Annual Mean Temperature**	0.38	< 0.10
**Growing Season Temperature**	0.61	< 0.01
**Livestock Density**	−0.44	< 0.10

Note: The growing season is defined as May–September. Associations with 0.05 < *p* < 0.10 were regarded as marginally significant. Positive *r* values indicate that higher values of the variable are associated with higher *D*_*I*_ (less severe desertification).

The four groups of 2D models exhibited the maximum classification accuracy for non-desertification and mild desertification, followed by moderate and severe desertification, with the lowest accuracy observed for extremely severe desertification. In the classification of non-desertification, the user accuracies for SA-SIF and SA-MSAVI were 0.9740 and 0.9231, respectively, whereas the user accuracies for SA-EVI and SA-NDVI were below 0.9000. The user accuracies for the classification of mild desertification were 0.8889 for SA-SIF and 0.8095 for SA-MSAVI, whereas SA-EVI and SA-NDVI exhibited user accuracies below 0.8000. The user accuracies for the classification of moderate desertification were 0.8571 for SA-SIF and 0.7912 for SA-MSAVI, whereas SA-EVI and SA-NDVI exhibited user accuracies below 0.7600. The user accuracies for the classification of severe desertification were 0.9000 for SA-SIF and 0.7885 for SA-MSAVI, whereas SA-EVI and SA-NDVI exhibited user accuracies below 0.7600. In the classification of extremely severe desertification, the user and producer accuracies for SA-SIF and SA-MSAVI were 0.8000/0.7111 and 0.7778/0.7778, respectively, whereas the user accuracies for SA-EVI and SA-NDVI were below 0.6500.

The overall accuracies of SA-SIF, SA-MSAVI, SA-EVI, and SA-NDVI were 0.8675, 0.8225, 0.7875, and 0.7725, respectively. The Kappa coefficient values for SA-SIF, SA-MSAVI, SA-EVI, and SA-NDVI were 0.8452, 0.7938, 0.7521, and 0.7346, respectively.

### 3.5. Hierarchical mapping of desertification utilizing the SA-SIF 2D model from 2001 to 2020

To quantitatively evaluate the drivers of the observed desertification trends, Pearson’s correlation analysis was performed between the annual average *D*_*I*_ and key climatic variables over the 2001–2020 period (Table 6). Annual precipitation showed a moderate positive correlation with average *D*_*I*_ (*r* = 0.52, *p* < 0.05), suggesting that increased precipitation is associated with reduced desertification severity. Annual mean temperature showed a weaker but still significant positive correlation (*r* = 0.38, *p* < 0.10). Growing season temperature exhibited the strongest correlation (*r* = 0.61, *p* < 0.01), consistent with the importance of thermal conditions for vegetation growth on the QTP. In terms of human activities, livestock density data (where available) showed a negative correlation with *D*_*I*_ (*r* = −0.44, *p* < 0.10), indicating that areas with higher grazing pressure tended to have lower *D*_*I*_ values (i.e., more severe desertification).

**Fig 5:**
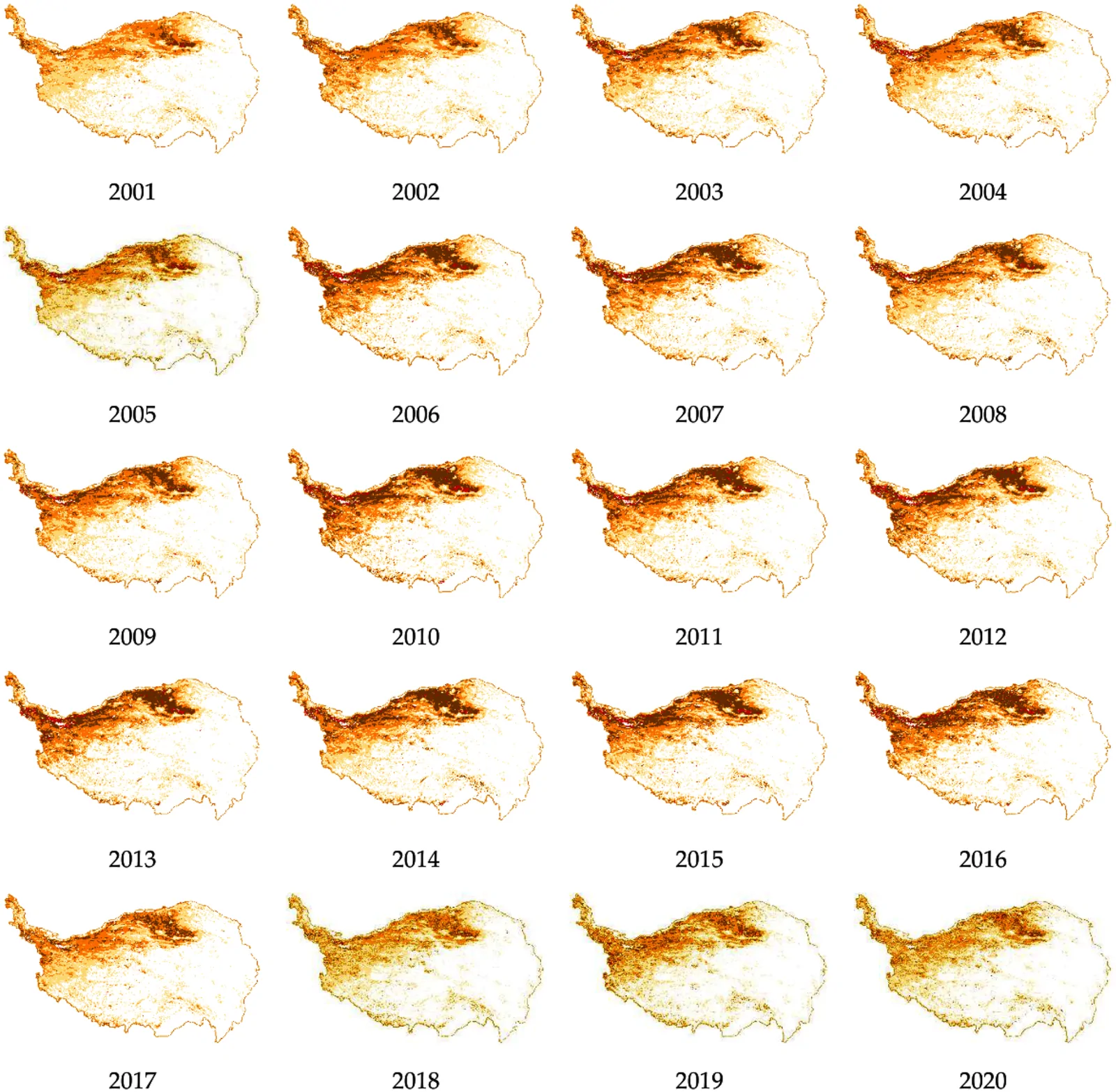
Classification mapping findings of desertification using the SA-SIF 2D model on the QTP from 2001 to 2020. The five levels of desertification correspond to those depicted in Fig 4 Maps were generated using ArcGIS 10.8.2 (https://www.esri.com/en-us/arcgis/). The basemap and MODIS data were obtained from the NASA LP DAAC (https://lpdaac.usgs.gov/). (Note: Due to space constraints in the 20-year series sub-map, only the desertification classification results are displayed in the figure. All other geographic annotation information remains consistent with Figs 1 and 4.).

**Fig 6:**
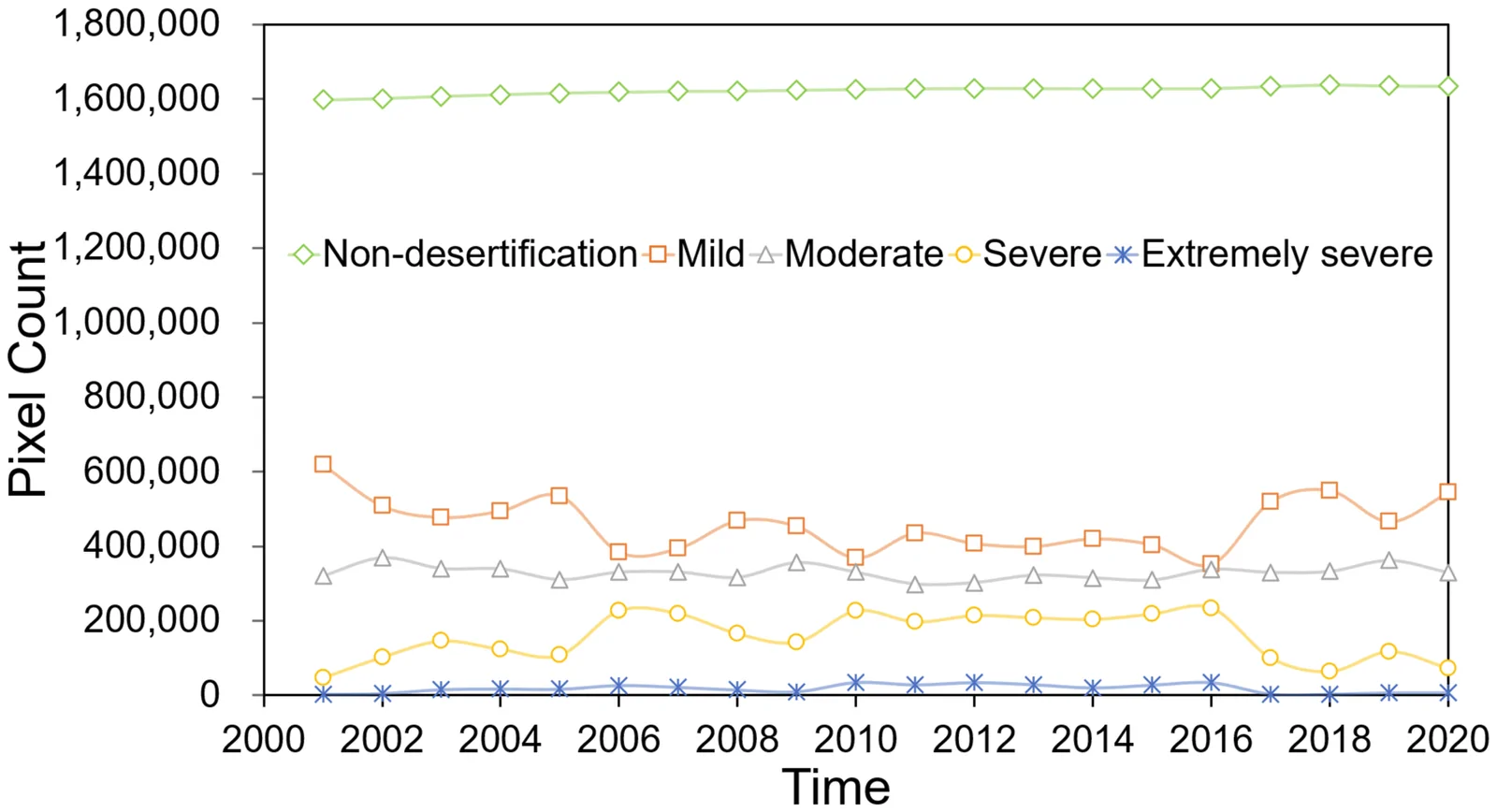
Dynamics of pixel counts for different desertification levels on the QTP from 2001 to 2020. The five levels of desertification were non-desertification, mild, moderate, severe, and extremely severe.

Fig 5 illustrates the outcomes of desertification categorization mapping of the QTP from 2001 to 2020. Fig 5 mirrors Fig 4 in categorizing desertification into five levels: non-desertification, mild, moderate, severe, and extremely severe. Fig 6 illustrates the trend in the number of desertification pixels across these five levels on the QTP from 2001 to 2020. Figs 5 and 6 were generated using the SA-SIF 2D desertification assessment model.

Figs 5 and 6 illustrate the spatial and temporal variations in desertification at all stages of the QTP from 2001 to 2020. (a) The peak incidence of extremely severe desertification was recorded in 2010, whereas the lowest incidence was noted in 2001. The tendency of severe desertification escalated in the first decade and diminished in the second. A considerable decline in extremely severe desertification was noted from 2016 to 2020. Regions of significant escalation and reduction of extremely severe desertification predominantly occurred in zones H and I. (b) The peak incidence of severe desertification was recorded in 2016, whereas the lowest incidence was noted in 2001. A pronounced upward trend in severe desertification was evident in the first decade, followed by a notable downward trend in the second decade. The most notable decline in severe desertification occurred between 2016 and 2020. The regions experiencing significant increases and declines in desertification were predominantly located in the northern parts of zones F, G, H, and I. (c) The peak level of moderate desertification was recorded in 2002, whereas the lowest level was noted in 2011. A minor declining trend in moderate desertification was observed in the first decade, followed by a slight increase in the second decade. The regions experiencing increases and declines in moderate desertification were primarily located in the northern sections of zones F and G, the western part of zone J, and zones H and I. (d) The peak incidence of mild desertification was recorded in 2001, whereas the lowest incidence was noted in 2016. A notable decline in mild desertification occurred in the first decade, followed by a considerable increase in the second. The most notable trend of mild desertification increase was recorded between 2016 and 2020. The regions of mild desertification rise and reduction predominantly occurred in the central part of zone G, the eastern part of zone J, and in zones E and F. (e) The peak value of non-desertification was recorded in 2018, whereas the lowest value was noted in 2001. The phenomenon of non-desertification exhibited a modest upward tendency over the two-decade span. Regions exhibiting growth and decline in non-desertification predominantly occurred in the central and eastern parts of the QTP.

## 4. Discussion

### 4.1. Model performance and comparison

In this study, four groups of 2D desertification assessment models (SA-NDVI, SA-MSAVI, SA-EVI, and SA-SIF) were developed. The SA-SIF model was implemented for the first time on the QTP and on a global scale. Among the four model groups, the SA-SIF model surpassed the other three in classification performance. The rankings for the overall accuracy and Kappa coefficient were SA-SIF > SA-MSAVI > SA-EVI > SA-NDVI. The overall accuracy and Kappa coefficient of the SA-SIF model exceeded those of the SA-NDVI model by 0.0950 and 0.1106, respectively. In the identification of severe desertification, the user accuracy increased from 0.7358 in the SA-NDVI model to 0.9000 in the SA-SIF model. The statistical results summarized in Tables 4–6 further corroborate these findings: the bootstrap confidence intervals confirm the stability of the SA-SIF model accuracy estimates, McNemar’s tests validate its significant superiority over the other models, and Pearson correlation analysis provides quantitative evidence linking *D*_*I*_ to climatic and anthropogenic drivers.

The user and producer accuracies of the SA-MSAVI and SA-SIF models surpassed those of the SA-NDVI and SA-EVI models in identifying various levels of desertification. The four model groups exhibited superior accuracy in identifying non-desertification and diminished performance in detecting extremely severe desertification. First, owing to the comparatively greater vegetation in non-desertification regions, NDVI, MSAVI, EVI, and SIF can proficiently identify vegetation. The MSAVI mitigates signal noise and soil background interference by utilizing soil conditioning factors [39,71,72] and effectively differentiates between soil and vegetation, even in regions with low vegetation cover. The SA-MSAVI model demonstrated enhanced accuracy in identifying non-desertification areas. As SIF is closely correlated with vegetation photosynthesis and has a heightened sensitivity to chlorophyll variations [41–43], SIF can be used to detect flora using fluorescence signals. Lu et al. discovered that the association between SIF and the photosynthetic activity of vegetation surpassed that of the conventionally structured vegetation index [73]. The SA-SIF model exhibited the highest accuracy in identifying non-desertification areas. Second, regions experiencing extremely severe desertification exhibited comparatively sparse vegetation. NDVI and EVI are challenging to use owing to insufficient data on vegetation. While MSAVI can manage soil, the presence of complicated components, such as saline and sandy gravels, increases following significant vegetation loss [74]. The proliferation of these materials induced spectral interference, diminishing the recognition efficacy of the SA-MSAVI model. Despite its sensitivity to chlorophyll fluctuations, SIF exhibited a diminished fluorescence signal in areas of acute desertification where vegetation was almost absent. This resulted in a diminished recognition capability of the SA-SIF model for extremely severe desertification. Furthermore, the elevated altitude of the QTP results in issues such as mountain shadows and atmospheric scattering [75], which diminish the accessibility of remote sensing signals across the four model groups to a certain degree.

Due to the intricate geographic conditions of the QTP, there is limited prior research employing the SA-NDVI/EVI/MSAVI/SIF model to directly assess desertification in the region. The only comparable analytical methodology was identified in the research conducted by Kuang et al. [76], who derived NDVI and surface data for the eastern QTP using Landsat imagery. Subsequently, they examined desertification in the eastern QTP. The overall accuracy of their experimental findings was 0.8205 [76]. To further assess the dependability of the experimental results of this investigation, the experimental findings of this study were contrasted with those from various study regions. The SA-NDVI model was developed from Landsat data by Ma et al. [63]. They examined desertification in Gaotai County, Gansu Province, utilizing the SA-NDVI model. The experimental findings exhibited an overall accuracy of 0.8407, with a Kappa coefficient of 0.7707 [63]; Wei et al. [77] utilized Landsat data to develop three model sets: SA-NDVI, SA-MSAVI, and SA-TGSI. The desertification in northwest Mongolia was examined by these three model groups. The SA-MSAVI model had an overall accuracy of 0.8560, representing the highest performance among the three model groups [77]; Xu et al. [78] similarly developed five model groups, specifically SA-NDVI, SA-MSAVI, SA-TGSI, TGSI-MSAVI, and TGSI-NDVI, utilizing Landsat data. The desertification of the Selenge River basin in Mongolia was examined using these five categories of models. The SA-MSAVI model had an overall accuracy of 0.8489, representing the highest performance among the five model groups [78].

The accuracy of the SA-MSAVI/SA-NDVI model in prior research was marginally superior to that of the SA-MSAVI/SA-NDVI model in this investigation; however, both were inferior to the accuracy of the SA-SIF model in this study. This is because the remote sensing data used in this study to gather vegetation and soil information differed from those used in other studies. Previous studies have benefited from smaller study areas and shorter observation periods. Consequently, researchers have utilized Landsat data with a spatial resolution of 30 m. The study region encompassed the entire QTP, which is characterized by its vast geography and prolonged observation duration. Consequently, MODIS/SIF data with a spatial resolution of 500 m were selected for this study. Incorporating high-resolution data, such as Landsat, into this study will improve the accuracy of the SA-SIF model in desertification analysis.

### 4.2. Interpretation of observed desertification dynamics and driving factors

The SA-SIF model was used to map the spatial distribution of desertification on the QTP from 2001 to 2020, facilitating an analysis of its developmental trends. The severity of desertification on the QTP decreased from northwest to southeast. According to the findings in Figs 5 and 6, severe and extremely severe desertification predominantly occurred in the northwestern regions H and I. These two regions are characterized by minimal precipitation, high evapotranspiration, and increased wind and sand activity. Non-desertification primarily occurred in regions A, B, C, and D in the eastern area. These regions exhibit increased vegetation density, warm and humid climates, and reduced wind and sand activity.

The year 2016 marked a critical turning point in the desertification classification of the QTP. Prior to 2016, extremely severe and mild desertification exhibited fluctuating changes. After 2016, extremely severe desertification significantly receded and transitioned to lower levels, whereas mild desertification notably increased. This divergent trend highlights a heterogeneous restoration trajectory across desertification grades.

The quantitative correlation analysis (Table 6) supports the interpretation that both climatic factors and human activities contributed to the observed desertification dynamics on the QTP. The significant positive correlation between *D*_*I*_ and growing season temperature (*r* = 0.61, *p* < 0.01) suggests that warming may have promoted vegetation growth and thereby reduced desertification severity, consistent with findings from previous studies [79]. The negative correlation between *D*_*I*_ and livestock density (*r* = −0.44, *p* < 0.10) points to the potential role of grazing pressure in driving degradation.

The turning point observed around 2016, after which extremely severe desertification receded substantially, coincides with the implementation of major ecological restoration policies [80]. Although correlation analysis cannot establish causation, the temporal alignment is suggestive and warrants further investigation through formal attribution studies. These interpretations remain hypotheses that require more rigorous testing using process-based models and higher-resolution data.

The core driving force behind this shift was the synergistic recovery of SA and vegetation. After 2016, the SIF markedly increased, directly reflecting enhanced vegetation photosynthetic activity and the leaf area index [79]. Improved vegetation cover led to a sustained decrease in SA, enhancing the soil water and heat retention capacity [81]. This created conditions for drought-tolerant vegetation growth in areas of extremely severe desertification, driving the transition from extremely severe desertification to lower levels. External climatic conditions also supported this shift. After 2016, the number of precipitation days in northern QTP increased substantially, significantly raising soil moisture in extremely severe desertification areas and meeting the water requirements for herbaceous vegetation growth [80]. Concurrently, the freeze-thaw period on the QTP has progressively shortened, reducing soil exposure time and wind erosion intensity, thereby effectively curbing desertification expansion [82]. Furthermore, the annual expansion of non-desertification areas has benefited from environmental policies implemented by local governments, including afforestation, soil and water conservation, sand fixation and prevention, and grazing restrictions [80,83,84]. Over the past decade, the overall trend of severe and extremely severe desertification on the QTP has shown signs of slowing. However, influenced by both climate fluctuations and human activities, the rates of mild and moderate desertification have not exhibited a sustained decline but have instead experienced significant periodic fluctuations. This suggests that the future trajectory of desertification on the QTP may become more complex, necessitating the application of new technologies and methodologies for in-depth research.

### 4.3. Limitations and future directions

Although the validation samples were derived from Landsat 7 imagery across the 2001–2020 study period, the reference interpretation for each year was based on the best available cloud-free imagery during the growing season to minimize atmospheric and cloud interference. This ensured that the validation reference data were representative of the typical desertification conditions for each year. Nevertheless, multisource validation data incorporating higher-resolution imagery or field survey data would further strengthen the robustness of long-term trend analysis in future studies. Previous studies have successfully employed multi-factor spatiotemporal GIS frameworks to evaluate distribution anomalies and risk hotspots under changing environmental conditions [85–87]. These methodological approaches can serve as useful references for developing more comprehensive validation and attribution strategies for QTPs.

Despite its superior performance, the SA-SIF model has some limitations that warrant further improvement. First, the spatial resolution of the data (500 m) may not capture fine-scale desertification features, and future studies should explore the integration of higher-resolution remote sensing data. Second, the model’s applicability to other ecosystems and climatic regions remains to be validated, and cross-regional comparative studies will help establish its generality. Third, the current model framework considers only albedo and vegetation indicators; incorporating additional environmental factors such as soil moisture, topography, and land use intensity could further enhance its predictive capability and interpretive power.

## 5. Conclusion

This study, by constructing four two-dimensional desertification assessment models (SA-NDVI, SA-MSAVI, SA-EVI, and SA-SIF), systematically addressed the core question of whether coupling vegetation indices with SA can more accurately monitor the spatiotemporal dynamics of desertification on the QTP. Compared with conventional structural vegetation index-based models, the SA-SIF assessment framework provides substantially improved classification accuracy. This superiority arises because SIF responds directly to vegetation photosynthetic physiology rather than merely reflecting greenness structure, enabling earlier and more sensitive detection of vegetation degradation signals. The spatial pattern revealed by this model, showing decreasing desertification severity from northwest to southeast and the marked contraction of extremely severe desertification around 2016, delivers independent evidence for evaluating regional ecological restoration effectiveness. Nevertheless, the model requires further refinement in spatial resolution, cross-regional generalizability validation, and integration of multiple environmental factors. Future efforts should focus on developing higher-resolution physiological-physical coupling models and employing formal attribution frameworks to quantify the relative contributions of climatic variability and anthropogenic activities to desertification processes.

## Supporting information

S1 Fig Spatial distribution of 400 validation points on the QTP. The validation points were generated using stratified random sampling in ArcGIS 10.8.2 (https://www.esri.com/en-us/arcgis/), with stratification based on desertification grade (five classes) and geographic sub-basin (ten major basins), and are displayed using uniform symbols. The basemap and MODIS data were obtained from the NASA LP DAAC (https://lpdaac.usgs.gov/). All other geographic annotation information remains consistent with Figs 1 and 4.
(TIF)

S1 Table *D*_*I*_ classification confusion matrix for SA-NDVI group. (PDF)

S2 Table *D*_*I*_ classification confusion matrix for SA-MSAVI group. (PDF)

S3 Table *D*_*I*_ classification confusion matrix for SA-EVI group. (PDF)

S4 Table *D*_*I*_ classification confusion matrix for SA-SIF group. (PDF)

S1 Data Sampling point coordinates and multi-year attribute data (2001–2020). Spatial coordinates and annual attribute data of 2,000 stratified random sampling points used for constructing the Surface Albedo–Vegetation Index feature space across the QTP. Includes longitude, latitude, basin code, desertification level, and annual values of NDVI, MSAVI, EVI, SIF, and surface albedo for each point from 2001 to 2020.
(XLSX)

S2 Data Geographic information of validation and training samples. Geographic coordinates of 400 validation points and 400 training samples used for accuracy assessment. Both sets were generated using stratified random sampling in ArcGIS 10.8.2 (https://www.esri.com/en-us/arcgis/), stratified by desertification grade (five classes) and ten major hydrological basins, with area-weighted allocation. The validation and training samples are spatially independent.
(XLSX)

S3 Data Power analysis and sample size justification. Statistical justification for the 400 validation sample size, including the sample size calculation formula, parameter settings, and required sample sizes under different confidence levels and margins of error. Demonstrates that 400 samples exceed the minimum requirement (*n* = 196 at 95% confidence level with ±5% precision) by more than twofold.
(XLSX)

S4 Data Surface Albedo–Vegetation Index feature space scatter plot data. Scatter plot data for the four Albedo–Vegetation Index feature space models (SA-NDVI, SA-MSAVI, SA-EVI, and SA-SIF) across the QTP from 2001 to 2020. Each sheet contains pixel-level values of surface albedo and the corresponding vegetation index for one model, used to construct the feature space and derive the desertification differentiation index (*D*_*I*_).
(XLSX)

S5 Data Desertification classification results of four models (2001–2020). Annual desertification classification results for the four models (SA-NDVI, SA-MSAVI, SA-EVI, and SA-SIF) across the QTP from 2001 to 2020. Includes pixel counts for five desertification levels (non-desertification, mild, moderate, severe, and extremely severe) for each year and each model, derived from the *D*_*I*_ using the natural breaks (Jenks) method.
(XLSX)

S6 Data Landsat-based validation accuracy of four models (2001–2020). Annual validation accuracy results of the four desertification classification models based on Landsat 7 high-resolution imagery interpretation. Includes OA and KC for each model from 2001 to 2020, validated using 400 stratified random sampling points with reference interpretations from the best available cloud-free Landsat 7 imagery for each year during the growing season.
(XLSX)

S7 Data Detailed statistical analysis procedures. Detailed calculation procedures and intermediate results for the three statistical analyses: (1) Bootstrap resampling with 1,000 iterations for 95% confidence intervals of OA and KC; (2) McNemar’s test with continuity correction for pairwise model comparisons; and (3) Pearson correlation analysis between annual mean desertification index and climatic/anthropogenic driving factors.
(XLSX)
