# Supplementary material for: Mapping and dynamic monitoring of desertification on the Qinghai-Tibetan Plateau using surface Albedo and Solar-Induced Chlorophyll Fluorescence
Source: PLoS One. 2026 Sep 25;21(9):e0359348. doi: 10.1371/journal.pone.0359348 (PMC13614598; doi:10.1371/journal.pone.0359348)
Supplement: Supplementary file 1 [file pone.0359348.s002.pdf]

S1 Table. D_I classification confusion matrix for SA-NDVI group.

D_I\Landsat 7	Non-Desertification	Mild	Moderate	Severe	Extremely Severe	Sum
**Non-Desertification**	68	7	2	1	0	78
**Mild**	5	65	8	6	2	86
**Moderate**	4	7	68	10	2	91
**Severe**	3	9	12	78	4	106
**Extremely Severe**	3	3	4	7	30	47
**Sum**	77	84	91	103	45	400
