# Supplementary material for: Mapping and dynamic monitoring of desertification on the Qinghai-Tibetan Plateau using surface Albedo and Solar-Induced Chlorophyll Fluorescence
Source: PLoS One. 2026 Sep 25;21(9):e0359348. doi: 10.1371/journal.pone.0359348 (PMC13614598; doi:10.1371/journal.pone.0359348)
Supplement: Supplementary file 2 [file pone.0359348.s003.pdf]

S2 Table. D_I classification confusion matrix for SA-MSAVI group.

D_I\Landsat 7	Non-Desertification	Mild	Moderate	Severe	Extremely Severe	Sum
**Non-Desertification**	72	5	1	0	0	78
**Mild**	3	68	6	5	2	84
**Moderate**	2	7	72	8	2	91
**Severe**	1	6	10	82	5	104
**Extremely Severe**	0	0	3	7	35	45
**Sum**	77	84	91	103	45	400
