# Supplementary material for: Mapping and dynamic monitoring of desertification on the Qinghai-Tibetan Plateau using surface Albedo and Solar-Induced Chlorophyll Fluorescence
Source: PLoS One. 2026 Sep 25;21(9):e0359348. doi: 10.1371/journal.pone.0359348 (PMC13614598; doi:10.1371/journal.pone.0359348)
Supplement: Supplementary file 3 [file pone.0359348.s004.pdf]

S3 Table. D_I classification confusion matrix for SA-EVI group.

D_I\Landsat 7	Non-Desertification	Mild	Moderate	Severe	Extremely Severe	Sum
**Non-Desertification**	70	6	1	1	0	78
**Mild**	4	66	7	6	2	85
**Moderate**	3	8	69	9	2	91
**Severe**	2	9	11	79	4	105
**Extremely Severe**	2	3	4	8	31	48
**Sum**	77	84	91	103	45	400
