# Supplementary material for: Mapping and dynamic monitoring of desertification on the Qinghai-Tibetan Plateau using surface Albedo and Solar-Induced Chlorophyll Fluorescence
Source: PLoS One. 2026 Sep 25;21(9):e0359348. doi: 10.1371/journal.pone.0359348 (PMC13614598; doi:10.1371/journal.pone.0359348)
Supplement: Supplementary file 4 [file pone.0359348.s005.pdf]

S4 Table. D_I classification confusion matrix for SA-SIF group.

D_I\Landsat 7	Non-Desertification	Mild	Moderate	Severe	Extremely Severe	Sum
**Non-Desertification**	75	2	0	0	0	77
**Mild**	2	72	4	3	0	81
**Moderate**	0	5	78	6	2	91
**Severe**	0	2	5	90	3	100
**Extremely Severe**	0	0	3	5	32	40
**Sum**	77	84	91	103	45	400
